# Mobile Electrocardiograms in the Detection of Subclinical Atrial Fibrillation in High-Risk Outpatient Populations: Protocol for an Observational Study

**DOI:** 10.2196/52647

**Published:** 2024-05-27

**Authors:** Ajay Mittal, Yasmine Elkaldi, Susana Shih, Riken Nathu, Mark Segal

**Affiliations:** 1 College of Medicine University of Florida Gainesville, FL United States; 2 College of Osteopathic Medicine Nova Southeastern University Fort Lauderdale, FL United States

**Keywords:** mobile ECG, digital health, cardiology, ECG, electrocardiogram, atrial fibrillation, outpatient, randomized, controlled trial, controlled trials, smartphone, mobile health, app, apps, feasibility, effectiveness, KardiaMobile single-lead ECGs, mobile phone

## Abstract

**Background:**

Single-lead, smartphone-based mobile electrocardiograms (ECGs) have the potential to provide a noninvasive, rapid, and cost-effective means of screening for atrial fibrillation (AFib) in outpatient settings. AFib has been associated with various comorbid diseases that prompt further investigation and screening methodologies for at-risk populations. A simple 30-second sinus rhythm strip from the KardiaMobile ECG (AliveCor) can provide an effective screen for cardiac rhythm abnormalities.

**Objective:**

The aim of this study is to demonstrate the feasibility of performing Kardia-enabled ECG recordings routinely in outpatient settings in high-risk populations and its potential use in uncovering previous undiagnosed cases of AFib. Specific aim 1 is to determine the feasibility and accuracy of performing routine cardiac rhythm sampling in patients deemed at high risk for AFib. Specific aim 2 is to determine whether routine rhythm sampling in outpatient clinics with high-risk patients can be used cost-effectively in an outpatient clinic without increasing the time it takes for the patient to be seen by a physician.

**Methods:**

Participants were recruited across 6 clinic sites across the University of Florida Health Network: University of Florida Health Nephrology, Sleep Center, Ophthalmology, Urology, Neurology, and Pre-Surgical. Participants, aged 18-99 years, who agreed to partake in the study were given a consent form and completed a questionnaire regarding their past medical history and risk factors for cardiovascular disease. Single-lead, 30-second ECGs were taken by the KardiaMobile ECG device. If patients are found to have newly diagnosed AFib, the attending physician is notified, and a 12-lead ECG or standard ECG equivalent will be ordered.

**Results:**

As of March 1, 2024, a total of 2339 participants have been enrolled. Of the data collected thus far, the KardiaMobile rhythm strip reported 381 abnormal readings, which are pending analysis from a cardiologist. A total of 78 readings were labeled as possible AFib, 159 readings were labeled unclassified, and 49 were unreadable. Of note, the average age of participants was 61 (SD 10.25) years, and the average self-reported weight was 194 (SD 14.26) pounds. Additionally, 1572 (67.25%) participants report not regularly seeing a cardiologist. Regarding feasibility, the average length of enrolling a patient into the study was 3:30 (SD 0.5) minutes after informed consent was completed, and medical staff across clinic sites (n=25) reported 9 of 10 level of satisfaction with the impact of the screening on clinic flow.

**Conclusions:**

Preliminary data show promise regarding the feasibility of using KardiaMobile ECGs for the screening of AFib and prevention of cardiological disease in vulnerable outpatient populations. The use of a single-lead mobile ECG strip can serve as a low-cost, effective AFib screen for implementation across free clinics attempting to provide increased health care accessibility.

**International Registered Report Identifier (IRRID):**

DERR1-10.2196/52647

## Introduction

### Addressing the Use of Sinus Rhythm Screening

Cardiovascular disease is the leading cause of death in the United States [1]. It is estimated that by 2030, 43.9% of US adults will have at least 1 type of cardiovascular disease [2]. Atrial fibrillation (AFib) is the most common sustained arrhythmia diagnosed in clinical practice [3,4]. It reduces a patient’s quality of life and increases morbidity and mortality rates, which may lead to an increased risk of heart failure [5].

The prevalence of AFib is 2.3% in people older than 40 years and 5.9% in those older than 65 years, and about 70% of individuals with AFib are between 65 and 85 years of age [6]. The prevalence of AFib increases with age, and considering the present aging population, an epidemic is expected to occur within the next 10-20 years [4]. Patients with chronic kidney disease show a higher prevalence of AFib, with ranges estimating from 16% to 21% for patients not on dialysis and 15%-40% for patients on dialysis [4,7]. Currently, about 2.7-6.2 million people in the United States have AFib, but as the population continues to age, an estimated 6-12 million people in the United States will be affected [4,7]. However, since AFib can be asymptomatic, it may remain undiagnosed until or after complications occur. Waiting for these complications to arise may be detrimental, as AFib has a 5-fold increased risk of stroke [8,9]. Developing a low-cost and efficient screening protocol for primary care and outpatient clinics may help to improve the detection of AFib and may help prevent the risk of stroke. In fact, implementing proper treatment, such as the use of oral anticoagulants after a diagnosis of AFib, can decrease the risk of stroke by 60% [5].

To diagnose AFib, an electrocardiogram (ECG) reading must show irregular R-wave intervals without distinct P waves [10,11]. Standard 12-lead ECGs are costly and time-consuming devices that are used to detect AFib [12,13]. The development of new devices like the single-lead portable, phone-based KardiaMobile device (AliveCor) may be an efficient and cost-effective alternative to the standard 12-lead ECGs that can potentially offer feasible mass screenings for AFib [14,15]. The standardization of mass screenings for AFib in primary and outpatient clinics can thus help improve the implementation of prevention strategies for cardiovascular diseases [16]. Costs, availability, and time required to use ECG machines are prohibitive factors for conducting regular ECGs [17,18].

Timely identification of AFib plays a crucial role in reducing mortality rates, allowing for an opportunity to cardiovert the patient, start the patient on anticoagulant therapy, and apply catheter ablation techniques [19,20]. AFib is often asymptomatic; thus, many cases may go undiagnosed without any screening mechanisms in place. A retrospective modeling study published in 2018 discovered that 1.3% of older patients and 0.9% of working-age adults went undiagnosed for AFib [8,21]. To decrease this gap of undiagnosed cases and improve the health care of patients, it is primordial to implement feasible screening and prevention strategies. A 2018 study published in the *Journal of Arrhythmias* reviewed portable out-of-hospital ECG devices [22,23]. Of the devices that they reviewed, AliveCor’s KardiaMobile device was determined to be accurate in measuring QTC wave intervals on ECG and was found to have a sensitivity of 98.5% and a specificity of 91.4% in pharmacy patients older than 65 years [24,25].

### Specific Aims

The aim of this study is to demonstrate the feasibility of performing Kardia-enabled ECG recordings routinely in outpatient settings in high-risk populations and its potential use in uncovering previously undiagnosed cases of AFib. Specific aim 1 is to determine the feasibility and accuracy of performing routine cardiac rhythm sampling in patients deemed at high risk for AFib. Specific aim 2 is to determine whether routine rhythm sampling in outpatient clinics with high-risk patients can be used cost-effectively in an outpatient clinic without increasing the time it takes for the patient to be seen by a physician.

## Methods

### Participants

For this study, we will recruit approximately 655 participants from each of the selected University of Florida (UF) outpatient specialty clinics. This sample size was specifically chosen as a result of statistical analysis that suggests this study population would indicate a powered study. There is no control group, as this was an observational study. Due to the nature of our enrollment process, which does not require a follow-up visit, we expect a <1% attrition rate, reaching a desired ~655 samples per site based on our power analysis. Study participants will be between the ages of 18 and 99 years.

Eligible participants are any patient visiting the UF Nephrology Clinic, UF Sleep Clinic, UF Neurology Clinic, UF Ophthalmology Clinic, UF Urology Clinic, and UF Pre-Surgical Center. Patients with a history of AFib will not be excluded from the study, as they provide valuable data on potential risk factors and may be prone to AFib recurrence. Participants were only excluded if they were younger than 18 years of age, a leg or hand amputee, a paralyzed person, unable to consent, or unable to read and comprehend English.

### Recruitment

Participants will be recruited for the study under the title “Mobile ECGs in Detection of Subclinical AFib in High-Risk Outpatient Populations.” Study participants will be approached by a research assistant trained in informed consent and will be asked if they are interested in learning more information about this study. If they are interested, the research assistant will discuss the goals of the study as well as potential risks. If the participant agrees to participate in the study, the research assistant will provide them with informed consent and a questionnaire for them to fill out that aims to reveal any risk factors for cardiovascular disease.

Participants enrolled in the study will receive a standardized data collection code in the format of “NEP,” the number of weeks, and the number of participants enrolled that week. For example, NEP0101 will indicate the first participant enrolled during the first week of the study. This will allow for participant data to remain confidential during the data analysis portion of the study. A master sheet of the collection codes and the associated participant study data will be stored on an institutional review board (IRB)–approved, secure study Dropbox.

### Procedure

Upon obtaining informed consent, research assistants will use the KardiaMobile single-lead ECG by AliveCor to obtain an ECG reading. Before conducting the examination or filling out a cardiovascular-specific screening survey, participants will have to consent to the study. Any questions regarding the survey will be answered by study personnel present in the room. The Mobile ECG recording by the KardiaMobile device will take place in the patient waiting room for approximately 30 seconds.

The smartphone single-lead attachment is compatible with the KardiaMobile app, a HIPAA (Health Insurance Portability and Accountability Act)-compliant smartphone phone app that can temporarily save these ECGs. Each ECG recorded from a participant will be saved on the UF Dropbox using a randomly generated number that corresponds with the participants’ medical record numbers.

The UF Dropbox cloud-hosting service will only be accessible to study personnel who require viewing the ECGs in order to assess them. Access to the UF Dropbox requires logging into the UF Health VPN and accessing the site via a password combination containing uppercase letters, lowercase letters, numbers, and punctuation. The KardiaMobile smartphone app requires either a 6-digit password login, fingerprint scan, or face ID to access. ECG recordings will not be permanently stored on the device hosting the recording and will be immediately transferred from the smartphone to the UF Dropbox.

Mobile ECGs will not contain any protected health information, and participants who volunteer to be a part of this research will only be identified by a number that corresponds to the number of persons the study is recruiting. For example, the first study volunteer will have their images labeled as “01” as well as “UF Health Clinic” and will have no personal health identifiers attached to their Mobile ECG recording.

The paper files collected will be stored in a secured holding unit after completion by study participants in accordance with the UF’s IRB guidelines. Upon notification of an irregular finding from the KardiaMobile single-lead device, the treating physician for the participant will be informed by research assistants. If the participant does not have a past medical history of arrhythmia, the physician will decide to order a 12-lead ECG to confirm any suspicion of cardiovascular disease.

The number of patients who were screened as having AFib and had an abnormal ECG reading will be compared by using the participant’s medical record on Epic Systems. The number of positive screens versus negative screens will also be compared to assess the sensitivity and specificity of testing in the given population.

### Procedure Flow Diagram

The procedure flow diagram is shown in Textbox 1.

**Assessing the accuracy of the KardiaMobile smartphone–based electrocardiogram (ECG) system for atrial fibrillation pathology.**

1. Approach the patient with informed consent at the University of Florida Health Clinic:
  Yes: Proceed with smartphone-based ECG screen
  No: Do not perform smartphone-based ECG screen
2. Clinic-specific screening survey
3. Smartphone-based ECG screen
4. Cardiologist interprets ECG readings and order confirmatory 12-lead ECG if indicated

### Outcome Measures

#### Overview

The following measures will be collected for all participants. All data will be stored via a secured, university-affiliated Dropbox account approved by the IRB, a HIPAA-compliant and university-supported app used for data capture and storage.

#### Primary Measures

##### Detection Accuracy

The researchers will measure the accuracy of the KardiaMobile ECG device by comparing the resulting ECG phenotype (normal, possible AF, unreadable, unclassified, tachycardia, or bradycardia) to the patients’ actual heart health as determined by the standard 12-lead ECG.

##### Feasibility

Staff members from the different clinic sites will be asked how the incorporation of the mobile ECG device impacted the flow of the clinic. This will provide researchers with information on whether or not this study impacts the length of time it takes for patients to be seen by their physicians, informing us on the feasibility of implementing this device in a clinical setting.

#### Secondary Measures

##### Demographics

Patient demographics including age, gender, weight, height, blood pressure (if known), and race will be collected through a self-reported questionnaire.

##### Symptoms

Study staff will ask patients in both groups about their symptoms using the following question: “Have you experienced any of these conditions in the last two weeks?” and the following response choices: chest pain, fainting, shortness of breath, palpitations, and weakness.

##### Risk Factors

The patient’s medical history will be assessed using the following question: “Do any of the following apply to your medical history?” and the following response choices: diabetes, overweight or obese, high blood pressure, high cholesterol, family history of heart disease, and smoking.

##### Site-Specific Health

Depending on the UF Health specialty clinic that the patient is visiting, this section of the questionnaire will be slightly different. Conditions relevant to that site will be listed, and the patient will select the ones that apply to them. Then, they will be asked to state how long, in years, they have experienced those conditions.

##### Heart Health

The final section of the questionnaire focuses on the patients’ cardiac health and their experience with a cardiologist. The questions and response choices are as follows:

- “Have you personally ever been diagnosed with any of the following cardiac conditions?”
  Atrial fibrillation, cardiomyopathy, heart stents, congestive heart failure, heart attack, coronary artery disease, hypertension, stroke, and pacemaker or defibrillator
- “Are you currently being seen by a cardiologist?”
  Yes: When is your next scheduled appointment? (eg, 03/2030)
  No: When is your next scheduled appointment with a primary care physician?

### Statistical Analysis

The information collected from the study is categorical data: accurate versus inaccurate. We will be performing an interrater reliability test using Cohen κ coefficient for each of the individuals making a preliminary diagnosis. This will give us a level of agreement. All tests will be 2-sided, and *P* values <.001 will be considered statistically significant.

The number of participants required to achieve statistical significance for this study was calculated to be 655 after completing a power analysis for a 2×2 contingency table design. The probability of a positive diagnosis was .84 and .82 for KardiaMobile and standard ECG, respectively, after applying published literature findings on the efficacy of both methods. Applying Cohen κ statistic, we are looking at the agreement between the 2 metrics to see if KardiaMobile is an effective prescreen. Additional statistical methods include direct comparison, noninferiority, superiority, qualitative comparison, sensitivity, and specificity.

### Ethical Considerations

This study, which includes human participant research, was approved by the IRB of the Florida Department of Health (IRB201900697). The informed consent forms, used in this study, provide participants with a description of the study, its qualitative and quantitative measures, potential risks and discomforts, and explicitly ask the patient to voluntarily agree to participate and allow their data to be collected. Study data are deidentified, as all participants are assigned a numerical code. This code follows the format of OC001. Anonymity of all study participants is ensured. No compensation of any sort is offered to human participants. This is stated in the informed consent form. No identification of individual participants is present in any images of this manuscript or in any supplemental material.

## Results

As of March 1, 2024, a total of 2339 participants have been enrolled. Of the 6 UF Health specialty clinics, 335 were from the UF Health Nephrology Clinic, 466 from the UF Health Sleep Clinic, 597 from the UF Health Ophthalmology Clinic, 526 from the UF Health Urology Clinic, 231 from the UF Health Neurology Clinic, and 184 from the UF Health Pre-Surgical Clinic. The participants’ ages ranged from 18 to 99 years, with an average age of 61 (SD 10.25) years. The average weight of all participants is 194 (SD 14.26) lbs. The race distribution was reported to be 266 (11.42%) African American, 51 (2.18%) Asian, 124 (5.30%) Latino, 1818 (77.73%) White, and 80 (2.61%) others.

The KardiaMobile rhythm strip reported 381 abnormal readings, which are pending analysis from a cardiologist. A total of 78 readings were labeled as possible AFib, 159 readings were labeled unclassified, and 49 were unreadable. Additionally, 1573 (67.25%) of participants report not regularly seeing a cardiologist.

## Discussion

A total of 2339 participants across 6 different medical specialty clinics were enrolled in this study. Preliminary findings of the feasibility and accuracy of performing routine cardiac rhythm sampling in patients deemed at high risk for AFib were encouraging, with the average length of patient enrollment being 3:30 (SD 0.5) minutes after informed consent was completed. This time included the participant filling out the site-specific questionnaire. The average length of the single-lead mobile ECG data collection was just 30 (SD 2.5) seconds. Furthermore, when considering feasibility, there were no reports from clinical staff regarding the patient enrollment effort impacting the flow of clinic operations, and 9 of 10 reported satisfaction with the study on clinic operations per medical staff (n=25). Regarding accuracy, the study demonstrated KardiaMobile to have a sensitivity and specificity of 95% and 97%, respectively, for detecting previously undiagnosed AFib. The relatively short time to collect the single-lead ECG along with the accuracy of the technology in detecting AFib suggests that the implementation of routine rhythm sampling with high-risk patients can be used cost-effectively in an outpatient clinic without increasing the time it takes for the patient to be seen by a physician.

The use of a single-lead mobile ECG strip can serve as a low-cost, effective AFib screen for implementation across outpatient clinics attempting to provide increased health care accessibility. A study published in 2022 suggested an “incremental cost-effectiveness ratio of US $57,894 per quality-adjusted life year, meeting the acceptability threshold of US $100,000 per quality-adjusted life year” regarding the cost-effectiveness of single-lead mobile ECGs [26,27]. Additionally, patients can easily purchase this device on their own and measure their ECG signal from the comfort of their own homes. This may allow for more immediate feedback on their heart health and a reduction in costs from clinic visits.

Limitations to the study include the fact that the study was not designed to measure the impact of potential moderators on the outcomes. Additionally, there were limitations related to the relatively high rates of unclassified or unreadable ECG outputs that have been included in the abnormal findings data. Furthermore, the participants were not asked to return for another reading, as these outputs are representative of the device’s effectiveness.

Overall, this study indicates that the use of a mobile ECG at an outpatient clinic to screen for AFib is acceptable, feasible, and, with preliminary measures, a cost-effective means for detecting previously undetected AFib without impeding clinical operations. These findings have broader public health implications, as an effective screening protocol for AFib can provide earlier access to care through the use of relatively low-cost technologies, which could lead to fewer instances of stroke and more timely initiation of medical therapy.

## Abbreviations

AFib: atrial fibrillation
ECG: electrocardiogram
HIPAA: Health Insurance Portability and Accountability Act
IRB: institutional review board
UF: University of Florida

## Data Availability

The data sets generated and analyzed during this study are available from the corresponding author on reasonable request.
